# Fees for Making Autopsies in Cases of Legal Investigation

**Published:** 1864-11

**Authors:** 


FEES FOR MAKING AUTOPSIES IN CASES OF LEGAL INVESTIGATION.—We are authorized to state, as the result of a decision of the Court of Suffolk County on the fees for autopsies when ordered by a coroner, that for an ordinary autopsy $30 will be allowed instead of $20—the old fee. But whenever there are circumstances of unusual responsibility, or involving much loss of time in testifying, etc., that a proportionate compensation shall be paid, up to the fee of the new fee table, viz., $50. This action has just been taken on the claim of a member of the Boston Medical Association for making an autopsy, wherein the new charge of $50 was handed in to the District Attorney.—*Boston Journal.*

